# Prognostic Signatures Based on Ferroptosis- and Immune-Related Genes for Cervical Squamous Cell Carcinoma and Endocervical Adenocarcinoma

**DOI:** 10.3389/fonc.2021.774558

**Published:** 2022-01-11

**Authors:** Chaoqun Xing, Huiming Yin, Zhi-Yong Yao, Xiao-Liang Xing

**Affiliations:** ^1^ School of Public Health and Laboratory Medicine, Hunan University of Medicine, Huaihua, China; ^2^ The First Affiliated Hospital of Hunan University of Medicine, Huaihua, China

**Keywords:** CESC, DEGs, ferroptosis, immune, prognosis

## Abstract

Cervical squamous cell carcinoma and endocervical adenocarcinoma (CESC) are among the most common malignancies of the female genital tract. Ferroptosis and immunity regulate each other and play important roles in the progression of CESC. The present study aimed to screen ferroptosis- and immune-related differentially expressed genes (FI-DEGs) to identify suitable prognostic signatures for patients with CESC. We downloaded the RNAseq count data and corresponding clinical information of CESC patients from The Cancer Genome Atlas database; obtained recognized ferroptosis- and immune-related genes from the FerrDb and ImmPort databases, respectively; and screened for suitable prognostic signatures using a series of bioinformatics analyses. We identified eight FI-DEGs (*CALCRL*, *CHIT1*, *DES*, *DUOX1*, *FLT1*, *HELLS*, *SCD*, and *SDC1*) that were independently correlated with the overall survival of patients with CESC. The prediction model constructed using these eight FI-DEGs was also independently correlated with overall survival. Both the sensitivity and specificity of the prediction model constructed using these eight signatures were over 60%. The comprehensive index of ferroptosis and immune status was significantly correlated with the immunity of patients with CESC. In conclusion, the risk assessment model constructed with these eight FI-DEGs predicted the CESC outcomes. Therefore, these eight FI-DEGs could serve as prognostic signatures for CESC.

## Introduction

Cervical cancer is the fourth most frequently diagnosed cancer and the fourth leading cause of cancer-related death in women (1). There were almost 600,000 new cases and 340,000 deaths worldwide in 2020 (1). The risk of death from cervical cancer in women in developing countries is 0.9%, which is higher than that in developed countries (0.3%) (2). Despite a series of advances in the prevention, screening, and treatment of cervical cancer, there has been no significant improvement in outcome (3, 4). The main treatments for patients with cervical cancer include surgery or postoperative concurrent chemoradiotherapy, such as T-cell therapy and checkpoint inhibitors (5). However, once local invasion and distant metastasis occur, the survival rate is significantly reduced, and complications significantly increase, which means that the efficacy of radiotherapy is diminished. Patients with metastatic or recurrent cervical cancer have a poor prognosis, with a 5-year overall survival (OS) rate of only 17% (2). Cervical squamous cell carcinoma and endocervical adenocarcinoma (CESC) account for approximately 15% of cancer deaths in women, with the second-highest mortality rate (6). Therefore, it is necessary to identify suitable signatures to predict the outcomes of patients with CESC.

Ferroptosis is a novel type of cell death that was first proposed by Dixon in 2012 (7). Ferroptosis can be activated by diverse physiological conditions and pathological stress (8). Numerous studies have demonstrated that dysregulation of ferroptosis is involved in several cancers, including CESC (9–12). Immunotherapy is a new type of cancer treatment that boosts the immune system. Immunotherapy has shown promising advantages in terms of treatment efficiency and long-term patient survival (13). Previous studies have documented that immune cells and immune-related factors can regulate ferroptosis, thus achieving the antitumor effects of immunotherapy. For example, Wang et al. found that CD8^+^ T cells induce ferroptosis by downregulating *SLC3A2* and *SLC7A11*. The induction of ferroptosis effectively improves the antitumor effects of immunotherapy, which suggests that immunotherapy may be achieved by regulating ferroptosis. Additionally, previous studies have indicated that ferroptosis can regulate the immune system. The lipid metabolites released by ferroptotic cells can exert immunomodulatory effects on adjacent immune cells and induce an immune response (14). Therefore, to improve cancer diagnosis and prognosis, it is necessary to investigate the comprehensive network of ferroptosis- and immune-related genes.

Based on the currently known ferroptosis- and immune-related genes, and open public databases like The Cancer Genome Atlas (TCGA), it is hypothesized that prognostic signatures associated with ferroptosis and immune response might help to distinguish subgroups of CESC patients with distinct ferroptosis and immune phenotypes and survival profiles.

## Material and Methods

### Data Acquisition and Processing

RNAseq counts data (3 controls and 304 cancers) and the corresponding clinical information (**Supplementary Table 1**) was obtained from TCGA database. DESeq2 package in R 3.6.2 was used to screen the differentially expressed genes (DEGs). We utilized the specific criteria as following padj < 0.05, |logFC| ≥ 2.0, and basemean > 500 to filter the DEGs.

### Generation of Ferroptosis-Related Differentially Expressed Genes and Immune-Related Differentially Expressed Genes

Recognized lists of ferroptosis and immune-related genes were obtained FerrDb (http://zhounan.org/ferrdb) and ImmPort database (http://immport.org), respectively. There were 259 ferroptosis-related genes (FR-Genes), including 108 ferroptosis driver genes, 69 ferroptosis suppressor genes, and 111 ferroptosis marker genes. There were 1,793 immune-related (IR-Genes).

### Development of the Comprehensive Index of Ferroptosis and Immune Status

The ferroptosis and/or immune-related DEGs were first subjected to univariate Cox regression. The genes significantly related to the OS of patients with CESC were filtered by least absolute shrinkage and selection operator (LASSO) Cox regression analysis. Then, the candidate DEGs were then filtered by multivariate Cox regression analysis. The filtered candidate signatures were used for the risk assessment model construction, as follows: Risk score = βgene1 * Expgene1 + βgene2 * Expgene2 + ··· + βgenen * Expgenen (15). The β value was obtained from the multivariate Cox regression analysis. The expression values were obtained by DESeq2 analysis. The comprehensive index of ferroptosis and immune status (CIFI) value of each patient was calculated by using the risk score of the patient subtracted by the minimum risk score of the cohort, which was then divided by the maximum risk score of the cohort, namely, CIFI = (Risk score − Min)/Max (15).

### Immune Profile Analysis

The ESTIMATE package in R software (3.6.2) was performed to calculate the stromal score, immune score, tumor purity, and ESTIMATE scores by using the genes expression data. We downloaded the calculated data of immune cell infiltration of patients with CESC from the Tumor Immune Estimation Resource (TIMER) and carried out correlation analysis for the ESTIMATE value and candidate prognosis signatures.

### Principal Component Analysis

Principal component analysis (PCA) was conducted to reduce dimension and visualize the CESC patients with different CIFI values by 3 ferroptosis-related DEGs (FR-DEGs), 6 immune-related DEGs (IR-DEGs), and 8 ferroptosis- and immune-related DEGs (FI-DEGs) filtered by univariate Cox, LASSO analysis, and multivariate Cox analysis.

### Statistical Analysis

A repeated-measures ANOVA followed by unpaired two-tailed Student’s t-test was used as indicated. All results are expressed as mean ± SEM.

## Results

### Identification of Candidate Prognostic Signatures

**Figure 1:**
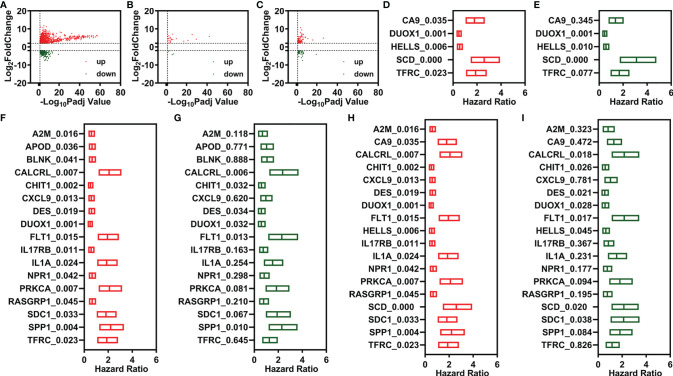
Identification of candidate prognostic signatures. **(A–C)** Volcano plot of DEGs, FR-DEGs, and IR-DEGs for CESC. **(D, E)** Univariate **(D)** and multivariate **(C)** Cox regression analyses illustrate 3 FR-DEGs independently associated with the OS of CESC. **(F, G)** Univariate **(F)** and multivariate **(G)** Cox regression analyses illustrate 6 IR-DEGs independently associated with the OS of CESC. **(H, I)** Univariate **(H)** and multivariate **(I)** Cox regression analyses illustrate 8 FI-DEGs independently associated with the OS of CESC. DEGs, differentially expressed genes; FR-DEGs, ferroptosis-related DEGs; IR-DEGs, immune-related DEGs; CESC, cervical squamous cell carcinoma and endocervical adenocarcinoma; OS, overall survival.

A total of 307 TCGA samples were included in the present study. The DESeq2 package in R 3.6.2 was used to screen the DEGs. There were 1,295 DEGs (969 upregulated and 326 downregulated) (**Figure 1A**). Of these, 25 were FR-DEGs (21 upregulated and 4 downregulated) (**Figure 1B**), and 129 were IR-DEGs (83 upregulated and 46 downregulated) (**Figure 1C**). Through univariate Cox regression and LASSO analysis, five FR-DEGs were significantly related to the OS of patients with CESC (**Figure 1D** and **Supplementary Figures 1A, B**). Then, multivariate Cox regression analysis of the five FR-DEGs was performed, and three FR-DEGs were determined to be independently related to the OS of patients with CESC (**Figure 1E**). Similarly, 6 out of 17 IR-DEGs were significantly independently related to the OS of patients with CESC (**Figures 1F, G** and **Supplementary Figures 1C, D**).

Additionally, we performed a comprehensive combined analysis of the 25 FR-DEGs and 129 IR-DEGs and found that 8 out of 18 FI-DEGs were independently related to the OS of patients with CESC (**Figures 1H, I** and **Supplementary Figures 1E, F**). The expression of these candidate prognostic signatures is shown in **Supplementary Figure 2**.

### Construction of the Comprehensive Index of Ferroptosis and Immune Status Classifier

**Figure 2:**
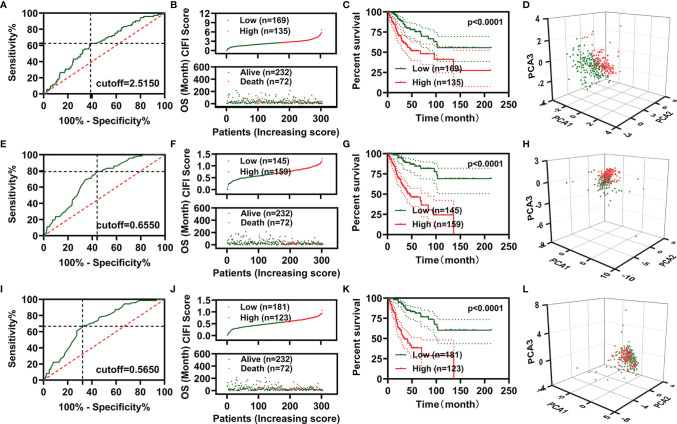
Development of prognostic signatures based on FR-DEGs, IR-DEGs, and FI-DEGs. **(A–D)** Optimal cutoff value **(A)**, distribution of the CIFI values (up) and survival status (down) **(B)**, overall survival curves **(C)**, and PCA plot **(D)** of the prognosis model constructed by three FR-DEGs. **(E–H)** Optimal cutoff value **(E)**, distribution of the CIFI values (up) and survival status (down) **(F)**, overall survival curves **(G)**, and PCA plot **(H)** of the prognosis model constructed by six IR-DEGs. **(I–L)** Optimal cutoff value **(I)**, distribution of the CIFI values (up) and survival status (down) **(J)**, overall survival curves **(K)**, and PCA plot **(L)** of the prognosis model constructed by eight FI-DEGs. DEGs, differentially expressed genes; FR-DEGs, ferroptosis-related DEGs; IR-DEGs, immune-related DEGs; FI-DEGs, ferroptosis- and immune-related DEGs; CIFI, comprehensive index of ferroptosis and immune status; PCA, principal component analysis.

The risk assessment scores were calculated by multiplying the gene expression of independent prognostic signatures and their corresponding coefficients, which were obtained by multivariate Cox regression analysis. A comprehensive index of ferroptosis and immune status (CIFI) values was calculated using the formula mentioned in the *Materials and Methods* section. The optimal cutoff value (Youden’s index) was used to regroup CESC patients (**Figures 2A, E, I**).

In the CIFI classifier constructed using the three FR-DEGs, 169 and 135 patients were classified as having high and low CIFI values, respectively. The CIFI values and survival time of the 304 patients in the model based on three FR-DEGs are displayed in **Figure 2B**. CESC patients with low CIFI values exhibited significantly better OS (**Figure 2C**). PCA indicated that CESC patients with low CIFI values could be clearly distinguished from those with high CIFI values (**Figure 2D**). We also constructed two more CIFI classifiers using the six IR-DEGs or eight FI-DEGs and performed the same analysis. Similar results were obtained for these two CIFI classifiers (**Figures 2E–L**).

**Figure 3:**
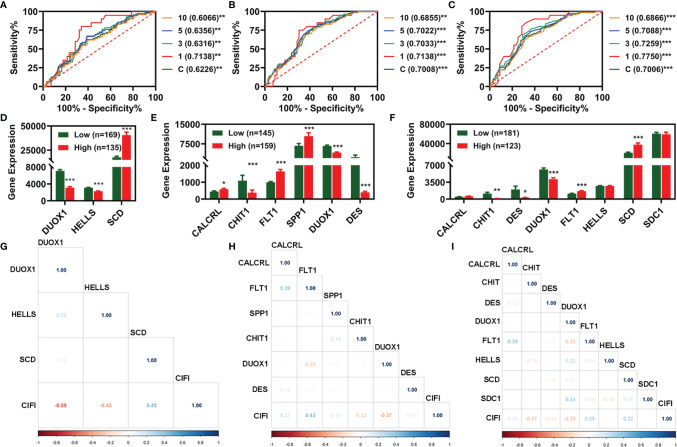
Evaluation of the three prognosis models constructed based on FR-DEGs, IR-DEGs, and FI-DEGs. **(A–C)**, ROC plot analysis for FR-DEG model **(A)**, IR-DEG model **(B)**, and FI-DEG model **(C)**. **(D–F)** The expression profile of the candidate prognosis biomarkers in different models. **(D)** FR-DEG model. **(E)** IR-DEG model. **(F)** FI-DEG models. **(G–I)** Correlation analysis of the CIFI value of the model with the candidate prognosis biomarkers. **(G)** FR-DEG model. **(H)** IR-DEG model. **(I)** FI-DEG models. *p < 0.05, **p < 0.01, ***p < 0.001. DEGs, differentially expressed genes; FR-DEGs, ferroptosis-related DEGs; IR-DEGs, immune-related DEGs; FI-DEGs, ferroptosis- and immune-related DEGs; ROC, receiver operating characteristic; CIFI, comprehensive index of ferroptosis and immune status.

We then plotted receiver operating characteristic (ROC) curves and found that the area under the curve (AUC) values of the two CIFI models constructed using the six IR-DEGs or eight FI-DEGs were comparable with each other and higher than those of the CIFI model constructed using the three FR-DEGs (**Figures 3A–C**). All AUC values of the three prognosis models at 1, 3, 5, and 10 years were over 0.6 (**Figures 3A–C**).

We compared the expression of candidate signatures between the high and low CIFI groups in the corresponding models (**Figures 3D–F**). The correlation analysis indicated that three FR-DEGs (*DUOX1*, *HELLS*, and *SCD*) were significantly correlated with the CIFI value of the FR model (**Figure 3G**), two IR-DEGs (*FLT1* and *DUOX1*) were significantly correlated with the CIFI value of the IR model (**Figure 3H**), and two FI-DEGs (*DUOX1* and *SCD*) were significantly correlated with the CIFI value of the FI model (**Figure 3I**).

### Independent Prognostic Value of the Comprehensive Index of Ferroptosis and Immune Status Classifier

**Figure 4:**
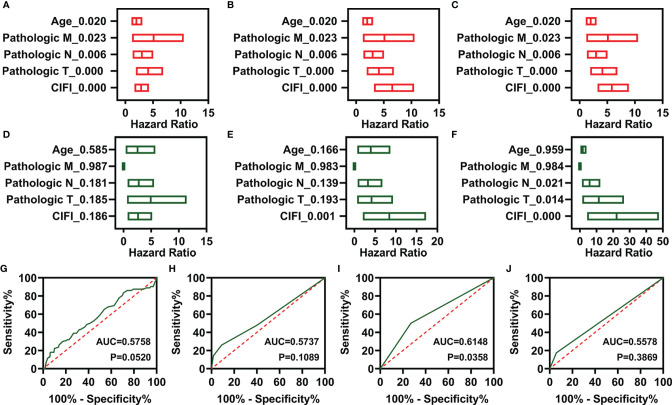
Independent prognostic factors of overall survival. **(A–C)** Univariate Cox regression of prognostic factors in three different models. **(A)** FR-DEG model. **(B)** IR-DEG model. **(C)** FI-DEG models. **(D–F)** Multivariate Cox regression of prognostic factors in three different models. **(D)** FR-DEG model. **(E)** IR-DEG model. **(F)** FI-DEG models. **(G–J)** ROC curve plot of CESC patients with different clinical features. **(G)** Age. **(H)** Pathological T. **(I)** Pathological N. **(J)** Pathological M. DEGs, differentially expressed genes; FR-DEGs, ferroptosis-related DEGs; IR-DEGs, immune-related DEGs; FI-DEGs, ferroptosis- and immune-related DEGs; ROC, receiver operating characteristic; CESC, cervical squamous cell carcinoma and endocervical adenocarcinoma.

To determine whether these CIFI models could serve as independent prognosis models for patients with CESC, clinical features (including age and pathological TNM) and the three CIFI models were used to perform univariate Cox regression analysis. As illustrated in **Figures 4A–C**, the clinical features and three CIFI models were significantly correlated with the OS of CESC patients (**Figures 4A–C**). Then, multivariate Cox regression analysis was performed, and the results indicated that the models based on six IR-DEGs or eight FI-DEGs were still correlated with OS (**Figures 4D–F**).

Moreover, the AUC values of the CIFI models based on six IR-DEGs or eight FI-DEGs were higher than those of the model based on clinical features (**Figures 3B, C**, **4G–J**). The AUC values for the three CIFI models were 0.6226, 0.7008, and 0.7006, with sensitivities of 0.6250, 0.7917, and 0.6667 and specificities of 0.6121, 0.5603, and 0.6767, respectively (**Table 1**). The sensitivity and specificity of the model based on eight FI-DEGs were higher than those of the model based on three FR-DEGs. The sensitivity of the model based on six IR-DEGs was the highest, but the specificity was less than 0.6. Comparatively, the CIFI model based on the eight FI-DEGs may be the most suitable. Therefore, we used only this model in the following analysis.

**Table 1:** Prognosis models for distinguishing patients with CESC from alive patients.

	FR-DEG CIFI model		IR-DEG CIFI model		FI-DEG CIFI model	
	Real death	Real alive	Real death	Real alive	Real death	Real alive
Predicted death	45	90	57	102	48	75
Predicted alive	27	142	15	130	24	157
Total	72	232	72	232	72	232
Correct	45	142	57	130	48	157
Sensitivity	0.6250		0.7917		0.6667	
Specificity		0.6121		0.5603		0.6767

CESC, cervical squamous cell carcinoma and endocervical adenocarcinoma; FR-DEG, ferroptosis-related differentially expressed gene; CIFI, comprehensive index of ferroptosis and immune status; IR-DEG, immune-related differentially expressed gene; FI-DEG, ferroptosis- and immune-related differentially expressed gene.

**Figure 5:**
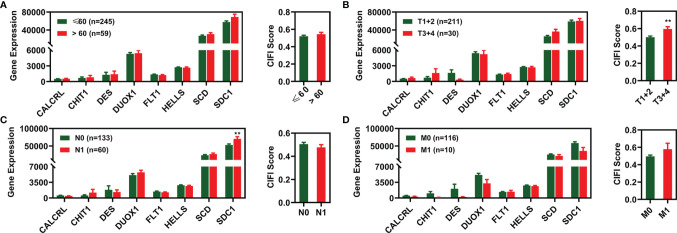
Expression profile of 8 FI-DEGs (left) and CIFI value (right) in different clinical features. **(A)** Age. **(B)** Pathological T. **(C)** Pathological N. **(D)** Pathological M. **p < 0.01. DEGs, differentially expressed genes; FI-DEGs, ferroptosis- and immune-related DEGs; CIFI, comprehensive index of ferroptosis and immune status.

We investigated how different clinical features were associated with the expression of candidate prognostic signatures and the CIFI values of the FI-DEG model. As illustrated in **Figure 5**, *SDC1* expression was significantly different between N0 and N1 patients with CESC. The CIFI values were significantly different between the T1+2 and T3+4 groups.

### Association Between Tumor Immune Infiltration and Comprehensive Index of Ferroptosis and Immune Status

**Figure 6:**
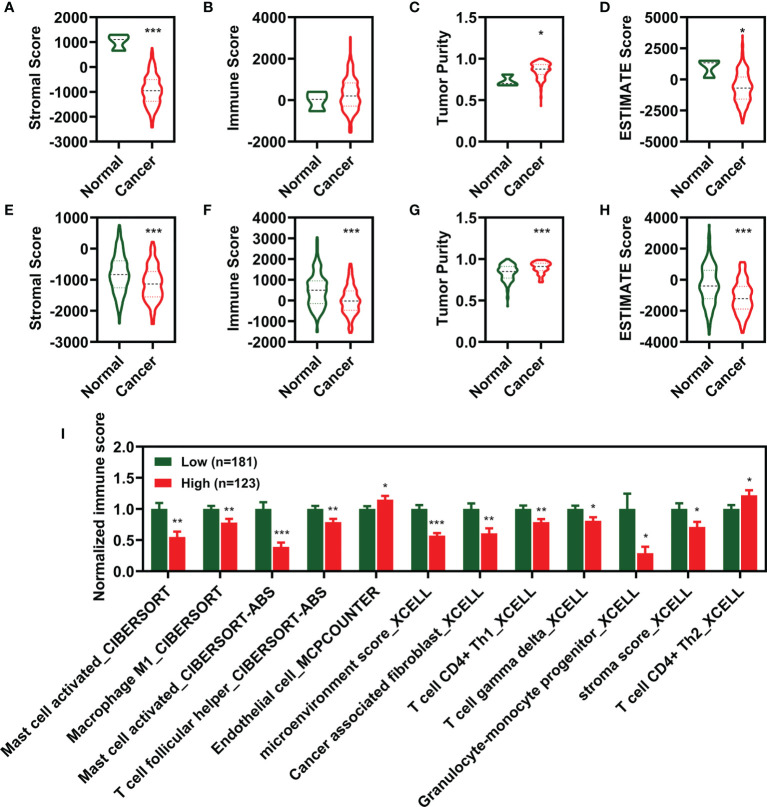
Estimation of the immunity in different groups. **(A–D)** Score of stromal **(A)**, Immune **(B)**, tumor purity **(C)**, and ESTIMATE **(D)** between normal and cancer. **(E–H)** Score of stromal **(E)**, Immune **(F)**, tumor purity **(G)**, and ESTIMATE **(H)** between low CIFI group and high CIFI group of eight FI-DEG models. **(I)** Identified immune molecules and factors verified by differential analysis between low CIFI group and high CIFI group. *p < 0.05, **p < 0.01, ***p < 0.001. CIFI, comprehensive index of ferroptosis and immune status; DEG, differentially expressed genes; FI-DEG, ferroptosis- and immune-related DEG.

**Figure 7:**
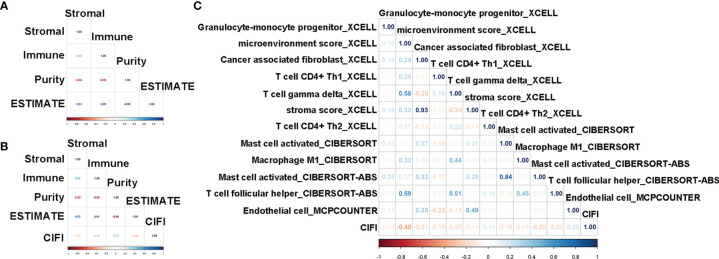
Correlation analysis of CIFI value with the ESTIMATE score and immune molecular and factors. **(A, B)** Correlation of the ESTIMATE scores with stromal, immune, and tumor purity in the sample of normal and CESC **(A)** in the CESC patients **(B)**. **(C)** Correlation of the CIFI value with different immune molecular and factors. CIFI, comprehensive index of ferroptosis and immune status; CESC, cervical squamous cell carcinoma and endocervical adenocarcinoma.

To characterize the relationship between the CIFI value and tumor immune infiltration, we utilized the ESTIMATE package in R 3.6.2 to calculate the stromal score, immune score, and tumor purity. As illustrated in **Figures 6A–D**, the stromal score, tumor purity, and ESTIMATE score were significantly different between the normal group and the patients with CESC. Moreover, the stromal score, immune score, and tumor purity were significantly correlated with the ESTIMATE score (**Figure 7A**).

Compared with CESC patients with low CIFI values, those with high CIFI values exhibited significantly decreased stromal scores, immune scores, and ESTIMATE scores, as well as significantly increased tumor purity (**Figures 6E–H**). The CIFI value was significantly correlated with the immune score, tumor purity, and ESTIMATE score (**Figure 7B**).

Since we initially examined the DEGs correlated with ferroptosis and immunity, we then investigated the correlation between CIFI and levels of tumor-infiltrating immune cells and immune-related factors. We found that the levels of 26 tumor-infiltrating immune cells and immune-related factors were different between the normal group and CESC patients (**Supplementary Table 2**), and 12 of the 26 were different between CESC patients with high and low CIFI values (**Figure 6I**). We found that the microenvironment score_XCELL was significantly correlated with CIFI (**Figure 7C**).

## Discussion

Cervical cancer is the fourth most frequently diagnosed cancer and the fourth leading cause of cancer-related deaths in women (1). Despite a series of advances in the prevention, screening, and treatment of cervical cancer, there has been no significant improvement in the improvement of cervical cancer outcomes (3, 4). Patients with metastatic or recurrent cervical cancer have a poor prognosis, with a 5-year OS rate of only 17% (2). Almost 15% of cervical cancers are CESC (6). Therefore, it is important to find suitable biomarkers that can predict the occurrence or prognosis of CESC. Previous studies have demonstrated that the dysregulation of ferroptosis and immunity could play an important role in the progression of CESC. In the present study, we aimed to screen FI-DEGs to identify suitable prognostic biomarkers for CESC. The prognosis model constructed using eight FI-DEGs performed better than the models constructed using three FR-DEGs or six IR-DEGs. The FI-DEG prognosis model independently predicted the outcome of CESC.

In CESC patients, the expression of *CHIT1*, *DUOX1*, *HELLS*, *SCD*, and *SDC1* was increased significantly, while that of *CALCRL*, *DES*, and *FLT1* was decreased significantly, compared with that in the normal group. Chitotriosidase (CHIT1), encoded by *CHIT1* gene, is a member of the chitinase family. Li et al. found that the variations rs61745299 and rs35920428 within the CDS region of *CHIT1* were associated with the risk of colorectal cancer (CRC). These two variations significantly increased the expression of *CHIT1* and were associated with CRC (16).

Dual oxidase 1 (DUOX1) is a member of the protein family of nicotinamide adenine dinucleotide phosphate oxidases. Previous evidence has shown that *DUOX1* is frequently downregulated in lung and liver cancers. However, Zhang et al. found that knockdown of *DUOX2* inhibited the invasion and migration of CRC cells by affecting the ubiquitination status of the ribosomal protein uL3 (17). In the present study, we found that *DUOX1* expression was increased in patients with CESC, which is consistent with Cho’s previous report (18). These results suggest that *DUOX1* may play different roles in different cancers, and it is also possible that the abnormal *DUOX1* expression was only the result of CESC, rather than the cause.

Helicase, lymphoid-specific (HELLS) is a chromatin remodeling enzyme. Overexpression of *HELLS* was correlated with more aggressive clinical–pathological features and poorer prognosis (19). In retinoblastoma, Zocchi et al. found that downregulation of *HELLS* could drastically reduce the ectopic division of differentiating cells in Rb1/p107-null retinae, significantly decrease the incidence of retinoblastoma, delay tumor progression, and increase OS (20). However, in pancreatic cancer, Hou found that *HELLS* is upregulated; downregulation leads to tumor growth arrest and increased sensitivity to cisplatin (21). Additionally, Zhu et al. found that *HELLS* levels are increased in lung cancer. Overexpression of *HELLS* is correlated with the prognosis of lung cancer, indicating that *HELLS* may be a potential diagnostic biomarker for lung cancer (22). In the present study, we found that *HELLS* expression was upregulated. Patients with high *HELLS* expression displayed a better OS. These results indicate that *HELLS* plays different roles in the progression of different cancers.

Stearoyl-CoA desaturase (SCD) is an enzyme that catalyzes the rate-limiting step in monounsaturated fatty acid synthesis. *SCD* is highly expressed in ovarian cancer tissues and cell lines and inhibits SCD-induced cell death (23). The results from the study by Tesfay indicated that SCD inhibition could be an effective component of antitumor therapy (23). Other studies have also shown that SCD can promote cancer cell proliferation, migration, and metastasis (24, 25).

SDC1 is a transmembrane heparan sulfate proteoglycan and a member of the syndecan proteoglycan family. Previous studies have demonstrated that SDC1 has different effects in different cancers. For example, reduced expression of *SDC1* could lead to advanced stages of gastric cancer and CRC, while increased expression could promote the growth and proliferation of pancreatic and breast cancers (26). Regardless of its inhibitory or activating effects, these studies indicate that SDC1 plays a pivotal role in cancer. Moreover, *SDC1* could serve as a prognostic biomarker for various cancers, such as hepatocellular carcinoma, gastric cancer, laryngeal cancer, and squamous cell lung carcinoma (27–29). In the present study, we found that *SDC1* was upregulated in CESC patients, and those with high expression of *SDC1* displayed worse OS. The correlation between *SDC1* expression and the OS of CESC patients was independent. Our results indicated that *SDC1* could be a diagnostic and prognostic biomarker for CESC.

Calcitonin receptor-like (CALCRL) is a G-protein-coupled neuropeptide receptor. CALCRL was increased in some tumor types, such as acute myeloid leukemia, Kaposi’s sarcoma, and Ewing sarcoma (30–32). High expression of *CALCRL* has been shown to be associated with poor prognosis in acute myeloid leukemia. Angenendt et al. found that knockout of *CALCRL* significantly impaired colony formation in human myeloid leukemia cell lines, indicating that CALCRL may be a potential therapeutic target in AML (33). Additionally, Larrue et al. found that CALCRL depletion reduces leukemic stem cell frequency in relapse-initiating cells post-chemotherapy *in vivo* (34). These results indicate that *CALCRL* is closely associated with cancer. In the present study, we found that *CALCRL* expression was decreased in CESC patients, although those with low *CALCRL* expression displayed a better OS. Although the results of our study are inconsistent with those of previous studies of other cancers, they further support a close relationship between *CALCRL* and cancer. Our results also suggest that CALCRL may play different roles in different cancers.

FLT-1, also known as vascular endothelial growth factor receptor 1 (VEGFR-1), is a high-affinity tyrosine kinase receptor for VEGF. Previous studies have demonstrated that *FLT1* expression is significantly correlated with OS in several cancers, including CRC, cholangiocarcinoma, and esophageal cancer (35–37). Additionally, Han et al. found that FLT1-rs9513111 was associated with a decreased risk of cervical cancer (38). Lv et al. found that the expression of *FLT1* was significantly correlated with the proliferation and invasion of trophoblast cells (39). FLT1 can inhibit proliferation, migration, and invasion of several cell types, such as CRC cells and trophoblast cells (39, 40). In the present study, we found that *FLT1* expression was independently correlated with the OS of CESC patients and could be used as a biomarker for CESC. Our present study reinforced that FLT1 may play an important role in the progression of CESC.

Arentz found that desmin (*DES*) expression was significantly increased in stage I and III tumors. In pancreatobiliary studies, Shin et al. found that *DES* could be used as a prognostic biomarker. We found that *DES* was downregulated in CESC patients. CESC patients with low *DES* expression had poorer survival than those with high expression.

## Conclusion

We found that the expression of eight FI-DEGs was independently correlated with the OS of CESC patients. The model constructed using these eight FI-DEGs was correlated with the OS of CESC patients and effectively predicted outcomes. These results indicated that the eight FI-DEGs may serve as prognostic and diagnostic biomarkers for CESC. However, further research is needed to determine how these genes affect CESC progression by ferroptosis and immunity regulation and whether they can be used for clinical prognosis.

## Data Availability Statement

The original contributions presented in the study are included in the article/**Supplementary Material**. Further inquiries can be directed to the corresponding author.

## Author Contributions

X-LX conceived and designed the experiments. CX and HY performed the analysis. Z-YY helped to analyze the data. X-LX wrote the paper. All authors contributed to the article and approved the submitted version.

## Funding

This project is financially supported by the Doctor Foundation of Hunan University of Medicine (2020122004), Hunan Provincial Science and Technology Department (2020SK51202), China Postdoctoral Science Foundation (2020TQ0365), and the Education Department of Hunan Province (20B417).

## Conflict of Interest

The authors declare that the research was conducted in the absence of any commercial or financial relationships that could be construed as a potential conflict of interest.

## Publisher’s Note

All claims expressed in this article are solely those of the authors and do not necessarily represent those of their affiliated organizations, or those of the publisher, the editors and the reviewers. Any product that may be evaluated in this article, or claim that may be made by its manufacturer, is not guaranteed or endorsed by the publisher.
