# Supplementary material for: Prognostic Signatures Based on Ferroptosis- and Immune-Related Genes for Cervical Squamous Cell Carcinoma and Endocervical Adenocarcinoma
Source: Front Oncol. 2022 Jan 11;11:774558. doi: 10.3389/fonc.2021.774558 (PMC8787259; doi:10.3389/fonc.2021.774558)
Supplement: Supplementary file 1 [file DataSheet_1.pdf]

Prognostic signatures based on ferroptosis- and immune-related genes for cervical squamous cell carcinoma and endocervical adenocarcinoma

Chaoqun Xing[1,2#], Huiming Yin[1,2#], Zhi-Yong Yao[1], Xiao-Liang Xing[1*]

[1]School of Public Health and Laboratory Medicine, Hunan University of Medicine, Huaihua 418000, Hunan, P. R. China.

[2]The first Affiliated Hospital of Hunan University of Medicine, Huaihua 418000, Hunan, P. R. China.

[#]contributed equally to this work.

[*]correspondence: Xiao-Liang Xing, xiaoliangxinghnm@126.com

Supplementary information: 2 figures and 2 tables

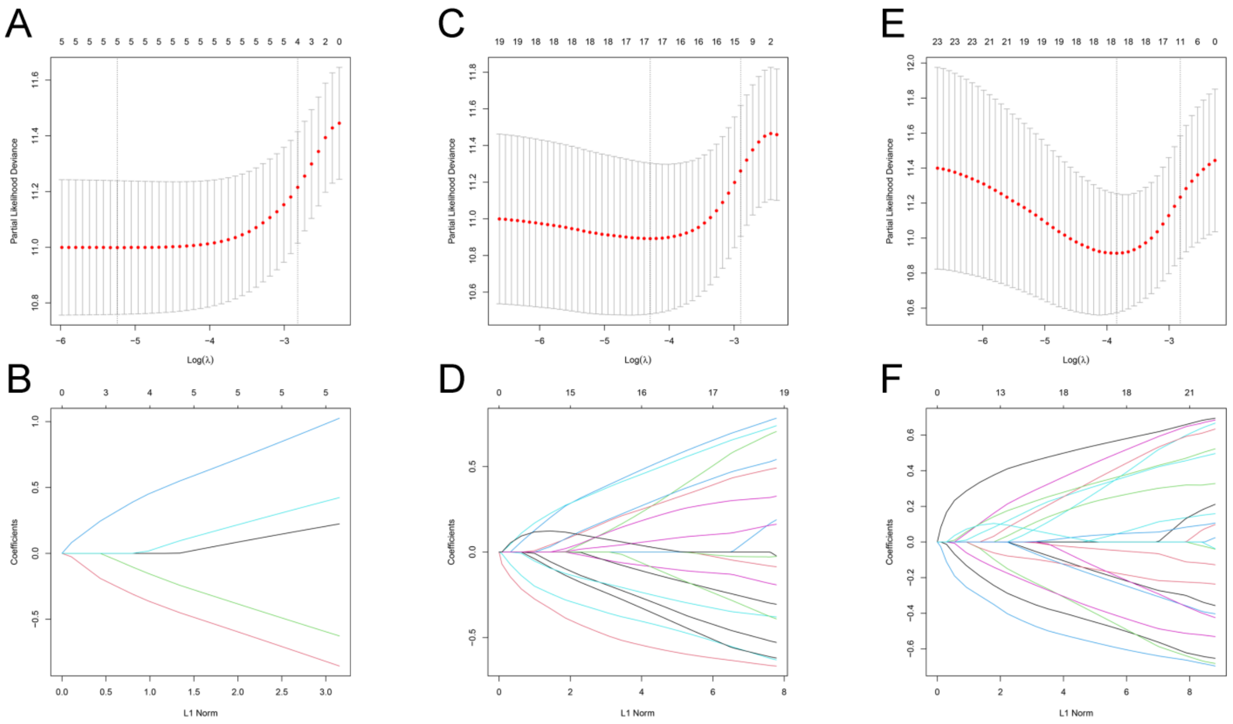

Figure 1 LASSO analysis for the candidate biomarkers verified by univatiate Cox regression. A-B, FR-DEGs, C-D, IR-DEGs, E-F, FI-DEGs.

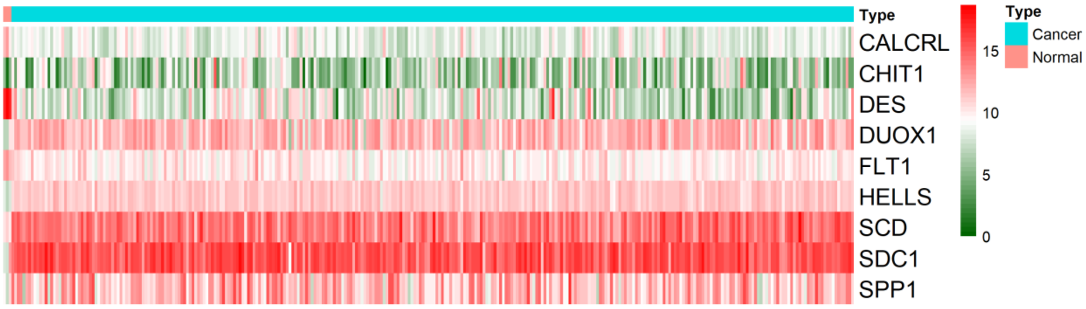

Figure 2 Heatmap of candidate FR-DEGs, IR-DEGs, and FI-DEGs between normal and cancer.

Table 1 Clinical characters of all patients used in present study.

case_submitter_id	vital_status	os	age	gender	pathologic		
					m	n	t
TCGA-C5-A8XJ	Alive	4467	74	female	NA	NA	NA
TCGA-EK-A3GM	Alive	0	65	female	NA	NA	NA
TCGA-VS-A957	Alive	1688	64	female	MX	NX	T1b1
TCGA-EK-A2RK	Alive	13	67	female	MX	N0	T1b1
TCGA-EK-A2RB	Alive	9	48	female	NA	NA	NA
TCGA-LP-A4AU	Alive	343	35	female	M0	N1	T2
TCGA-BI-A0VR	Alive	1505	53	female	M0	N1	T2b
TCGA-DS-A5RQ	Alive	512	80	female	MX	N0	T1b1
TCGA-JW-A5VK	Alive	623	43	female	MX	NX	T1b2
TCGA-VS-A950	Alive	1221	42	female	MX	NX	T3a
TCGA-JX-A5QV	Alive	636	37	female	M0	N0	T1b1
TCGA-EK-A3GN	Alive	27	47	female	NA	NX	TX
TCGA-EK-A2PG	Alive	46	88	female	NA	NA	NA
TCGA-DG-A2KM	Alive	1946	46	female	M0	N1	T1b1
TCGA-EK-A2RO	Alive	2	59	female	NA	NA	NA
TCGA-FU-A3WB	Alive	491	43	female	MX	N0	T2a2
TCGA-VS-A9U5	Alive	1535	57	female	MX	NX	T2b
TCGA-MY-A5BD	Alive	1667	62	female	M0	N0	T2b
TCGA-C5-A2LY	Alive	2383	30	female	M0	N0	T1b1
TCGA-FU-A5XV	Alive	321	32	female	MX	N1	T1b2
TCGA-JW-A5VJ	Alive	652	56	female	MX	NX	T2b
TCGA-VS-A952	Alive	1778	66	female	MX	N0	T1b
TCGA-EK-A2GZ	Alive	383	64	female	NA	NA	NA
TCGA-LP-A7HU	Alive	406	53	female	M0	N0	T2
TCGA-C5-A2LT	Alive	2226	38	female	M0	N0	T1b
TCGA-MA-AA42	Alive	259	75	female	M0	N0	T2b
TCGA-FU-A3EO	Alive	490	55	female	MX	N0	T2b
TCGA-LP-A4AV	Alive	0	63	female	M0	N0	T1b
TCGA-IR-A3LH	Alive	2394	49	female	M0	N0	T2a1
TCGA-VS-A9UD	Alive	739	73	female	MX	NX	T3a
TCGA-C5-A8XK	Alive	3039	30	female	NA	NA	NA
TCGA-EK-A2H1	Alive	799	20	female	NA	NA	NA

TCGA-EA-A3HS	Alive	959	35	female	M0	N0	T1b
TCGA-Q1-A73S	Alive	688	33	female	MX	N1	T1b1
TCGA-MA-AA3Z	Alive	595	43	female	M0	N0	T1b2
TCGA-EX-A1H5	Alive	619	58	female	MX	N1	T2b
TCGA-MA-AA3W	Alive	685	54	female	M0	N1	T1b1
TCGA-EX-A449	Alive	447	42	female	M1	NX	T1b1
TCGA-MU-A51Y	Alive	854	27	female	MX	N0	T2a1
TCGA-JW-A5VG	Alive	834	35	female	MX	NX	T2a
TCGA-FU-A3TX	Alive	45	78	female	M0	N0	T1b2
TCGA-VS-A9U7	Alive	1472	30	female	M0	N1	T1b
TCGA-EA-A3HT	Alive	954	68	female	M0	N0	T1b
TCGA-BI-A20A	Alive	720	49	female	M0	N0	T1b1
TCGA-VS-A9V3	Alive	540	62	female	MX	NX	T4
TCGA-EA-A411	Alive	747	50	female	M0	N0	T1b1
TCGA-ZJ-AB0I	Alive	0	25	female	MX	N1	T2b
TCGA-C5-A3HE	Alive	548	44	female	M0	N0	T1b2
TCGA-C5-A3HL	Alive	621	76	female	MX	N0	T1b2
TCGA-C5-A907	Alive	448	47	female	NA	NA	NA
TCGA-JW-A5VI	Alive	747	45	female	MX	NX	T2b
TCGA-VS-A94W	Alive	1243	39	female	MX	N1	T2b
TCGA-ZJ-AAXA	Alive	43	64	female	MX	N0	T1b
TCGA-ZJ-AB0H	Alive	0	48	female	M0	N1	T3b
TCGA-LP-A4AX	Alive	380	45	female	M0	N0	T1b1
TCGA-DG-A2KH	Alive	34	25	female	M0	N0	T1b1
TCGA-EX-A3L1	Alive	463	32	female	M0	N0	T2a1
TCGA-ZJ-AAXN	Alive	0	34	female	M0	N1	T1b2
TCGA-FU-A23L	Alive	725	60	female	MX	N0	T2a1
TCGA-VS-A9U6	Alive	1320	52	female	M1	NX	T4
TCGA-C5-A7X8	Alive	83	35	female	M0	N1	T2a
TCGA-ZJ-AAXF	Alive	0	62	female	M0	N0	T2b
TCGA-EA-A43B	Alive	791	43	female	M0	N0	T1b1
TCGA-EK-A2RN	Alive	71	45	female	MX	N1	T2b
TCGA-R2-A69V	Alive	596	42	female	M0	N0	T1b
TCGA-C5-A905	Alive	4879	37	female	MX	N0	T1b
TCGA-C5-A7CG	Alive	6408	55	female	MX	N0	T1b

TCGA-EX-A1H6	Alive	241	38	female	MX	N0	T1b1
TCGA-JW-A5VL	Alive	474	37	female	MX	NX	T1b2
TCGA-PN-A8MA	Alive	90	43	female	M0	N0	T2b
TCGA-ZJ-AAX4	Alive	21	85	female	M0	N0	T2
TCGA-HM-A4S6	Alive	454	51	female	MX	N1	T1b2
TCGA-FU-A3HY	Alive	954	47	female	MX	N1	T1b2
TCGA-C5-A1MP	Alive	109	34	female	MX	N1	T1b2
TCGA-EA-A44S	Alive	369	31	female	M0	N1	T2a2
TCGA-DS-A1OC	Alive	376	47	female	M0	N1	T1b1
TCGA-ZJ-AAXJ	Alive	0	43	female	MX	N0	T2b
TCGA-DR-A0ZL	Alive	2669	53	female	M0	N0	T1b1
TCGA-FU-A3HZ	Alive	1103	64	female	M0	N0	T2a2
TCGA-LP-A5U3	Alive	25	40	female	M0	N0	T1b1
TCGA-IR-A3LC	Alive	3935	40	female	M0	N0	T1b1
TCGA-C5-A1M7	Alive	1409	37	female	MX	N0	T1b
TCGA-EK-A2PM	Alive	18	81	female	NA	NA	NA
TCGA-VS-A9UZ	Alive	2044	61	female	MX	N0	T1b
TCGA-JW-AAVH	Alive	552	46	female	M0	N0	T1b1
TCGA-VS-A8QA	Alive	1099	44	female	MX	N0	T1b1
TCGA-C5-A8XI	Alive	254	69	female	MX	N0	T1b2
TCGA-DS-A1OD	Alive	3874	49	female	M0	N0	T1b1
TCGA-C5-A2LS	Alive	1345	37	female	M0	N0	T1b2
TCGA-FU-A40J	Alive	426	38	female	MX	N1	T2a1
TCGA-UC-A7PI	Alive	2114	44	female	MX	N0	T1b1
TCGA-4J-AA1J	Alive	542	31	female	M0	N0	T1b2
TCGA-ZJ-A8QO	Alive	0	73	female	NA	NA	NA
TCGA-VS-A8EH	Alive	986	56	female	M0	NX	T3b
TCGA-C5-A901	Alive	518	44	female	MX	N1	TX
TCGA-DS-A3LQ	Alive	699	46	female	M0	N1	T3b
TCGA-ZJ-AAXU	Alive	5	51	female	M0	N0	T2b
TCGA-ZJ-AAXT	Alive	0	54	female	M0	N1	T3b
TCGA-EK-A2PI	Alive	586	44	female	NA	NA	NA
TCGA-EK-A2RA	Alive	1246	74	female	MX	N0	T2a2
TCGA-EK-A2H0	Alive	1847	24	female	NA	NA	NA
TCGA-EA-A5ZF	Alive	828	56	female	M0	NX	T1b1

TCGA-C5-A2LV	Alive	2234	36	female	MX	N1	T1b
TCGA-C5-A1BK	Alive	5385	36	female	MX	N0	T1b
TCGA-C5-A7CO	Alive	4482	68	female	NA	NA	NA
TCGA-VS-A9UT	Alive	482	72	female	MX	N1	T1b1
TCGA-MA-AA41	Alive	279	33	female	M1	N1	T2
TCGA-VS-A954	Alive	1714	67	female	MX	NX	T3b
TCGA-ZJ-AAX8	Alive	0	58	female	M0	N1	T3b
TCGA-C5-A1BL	Alive	5271	32	female	NA	NA	NA
TCGA-C5-A1BJ	Alive	4385	34	female	NA	NA	NA
TCGA-C5-A1MQ	Alive	1031	35	female	M0	N0	T2b
TCGA-VS-A9V0	Alive	573	58	female	MX	NX	T1b
TCGA-EK-A2IR	Alive	3442	48	female	MX	N0	T1b
TCGA-C5-A902	Alive	149	35	female	NA	NA	NA
TCGA-VS-A9UC	Alive	825	32	female	MX	NX	T2b
TCGA-VS-A9UH	Alive	1427	53	female	MX	NX	T4
TCGA-MA-AA3X	Alive	617	50	female	MX	NX	T3b
TCGA-EA-A3Y4	Alive	1122	40	female	M0	N0	T1b
TCGA-MY-A5BE	Alive	1066	42	female	MX	N0	T1b1
TCGA-EK-A2RJ	Alive	53	51	female	MX	N0	T1b2
TCGA-VS-A8Q9	Alive	1630	79	female	MX	NX	TX
TCGA-MY-A5BF	Alive	634	68	female	MX	N0	T2a2
TCGA-EA-A3HQ	Alive	1136	60	female	M0	N0	T2a
TCGA-Q1-A6DV	Alive	491	36	female	M0	N0	T1b1
TCGA-EK-A3GJ	Alive	3	51	female	NA	N0	T1b1
TCGA-VS-A8EL	Alive	1992	38	female	M0	NX	T2b
TCGA-C5-A7XC	Alive	1551	26	female	M0	N0	T1b
TCGA-EA-A6QX	Alive	730	49	female	M0	N1	T1b1
TCGA-ZJ-AAXB	Alive	0	42	female	M0	N0	T1b2
TCGA-EA-A4BA	Alive	755	49	female	M0	N0	T1b2
TCGA-VS-A959	Alive	1561	76	female	MX	NX	T2b
TCGA-MA-AA43	Alive	346	48	female	MX	NX	T3b
TCGA-Q1-A5R1	Alive	474	32	female	MX	N0	T1b1
TCGA-EK-A2RM	Alive	50	40	female	NA	NA	NA
TCGA-EK-A2RE	Alive	57	26	female	MX	N0	T2a2
TCGA-RA-A741	Alive	444	34	female	NA	NA	NA

TCGA-FU-A23K	Alive	372	28	female	MX	N1	T1b1
TCGA-VS-A9V2	Alive	555	29	female	MX	N0	T1b
TCGA-EA-A439	Alive	965	50	female	M0	N0	T2a1
TCGA-C5-A7CH	Alive	4694	43	female	NA	NA	NA
TCGA-C5-A1M8	Alive	919	43	female	MX	N0	T1b
TCGA-VS-A8EG	Alive	1386	36	female	M0	NX	T1b1
TCGA-EK-A2PK	Alive	12	43	female	NA	NA	NA
TCGA-VS-A8EC	Alive	1415	55	female	M0	NX	T3b
TCGA-FU-A57G	Alive	1078	49	female	MX	N0	T1b2
TCGA-EK-A2R9	Alive	4	58	female	NA	N0	T1b1
TCGA-EK-A2RC	Alive	129	33	female	NA	NA	NA
TCGA-VS-A9UO	Alive	1456	43	female	MX	N0	T2b
TCGA-MY-A913	Alive	524	28	female	M1	N0	Tis
TCGA-VS-A94Z	Alive	1015	38	female	MX	NX	T2b
TCGA-DR-A0ZM	Alive	1791	61	female	M0	N0	T2b
TCGA-VS-A8QF	Alive	1800	42	female	MX	NX	TX
TCGA-VS-A958	Alive	1525	46	female	MX	NX	T2b
TCGA-DS-A7WH	Alive	533	34	female	MX	N0	T1b1
TCGA-EA-A3HR	Alive	940	57	female	M0	N0	T2a
TCGA-EA-A1QT	Alive	1243	47	female	M0	N0	T1b
TCGA-C5-A8ZZ	Alive	636	41	female	NA	NA	NA
TCGA-HM-A6W2	Alive	287	34	female	M1	N0	T2a
TCGA-C5-A7CM	Alive	619	35	female	NA	NA	NA
TCGA-MA-AA3Y	Alive	542	48	female	M0	N0	T1b1
TCGA-C5-A2LX	Alive	2526	54	female	MX	N1	T2
TCGA-EK-A2R8	Alive	44	48	female	MX	N1	T1b2
TCGA-DG-A2KJ	Alive	2893	50	female	M0	N1	T1b
TCGA-LP-A4AW	Alive	27	52	female	M0	N0	T1b
TCGA-IR-A3LF	Alive	2949	64	female	M0	N0	T1b1
TCGA-EA-A3QD	Alive	397	59	female	M0	N1	T1b1
TCGA-2W-A8YY	Alive	533	51	female	M0	N0	T2a2
TCGA-C5-A7UE	Alive	4738	45	female	NA	NA	NA
TCGA-C5-A7UH	Alive	3988	55	female	NA	NA	NA
TCGA-C5-A2M1	Alive	1169	37	female	MX	N0	T1b1
TCGA-EK-A3GK	Alive	15	33	female	MX	N0	T1b1

TCGA-DG-A2KL	Alive	1367	53	female	M0	N0	T2a
TCGA-MU-A5YI	Alive	1053	60	female	MX	NX	T1a1
TCGA-MU-A8JM	Alive	607	46	female	MX	N0	T1b
TCGA-DS-A0VM	Alive	3589	51	female	M0	N1	T1b1
TCGA-VS-A9UB	Alive	911	54	female	MX	N0	T2b
TCGA-Q1-A73R	Alive	567	45	female	MX	NX	TX
TCGA-FU-A2QG	Alive	579	29	female	NA	N0	T1b1
TCGA-C5-A1MF	Alive	1617	49	female	MX	N0	T1b2
TCGA-C5-A3HD	Alive	1582	51	female	NA	NA	NA
TCGA-ZJ-AAXI	Alive	0	67	female	M0	NX	T2b
TCGA-BI-A0VS	Alive	1735	48	female	M0	N0	T1b1
TCGA-EA-A5FO	Alive	815	59	female	M0	N0	T1b1
TCGA-LP-A5U2	Alive	9	30	female	MX	N1	T2a
TCGA-Q1-A5R3	Alive	485	56	female	MX	NX	TX
TCGA-EA-A97N	Alive	11	38	female	M0	N0	T1b2
TCGA-IR-A3LA	Alive	4172	60	female	M0	N0	T1b1
TCGA-JX-A3Q8	Alive	1357	40	female	MX	N0	T1b1
TCGA-FU-A3YQ	Alive	861	35	female	MX	N0	T1b1
TCGA-IR-A3LI	Alive	2493	48	female	M1	N1	T2b
TCGA-EA-A78R	Alive	410	54	female	M0	NX	T1b1
TCGA-JW-A69B	Alive	863	44	female	MX	NX	T1b2
TCGA-EA-A3QE	Alive	761	45	female	M0	N0	T1b
TCGA-VS-A9UR	Alive	793	53	female	MX	NX	T2a
TCGA-FU-A770	Alive	34	33	female	MX	N1	T2a2
TCGA-EA-A5ZD	Alive	830	40	female	M0	N0	T1b1
TCGA-ZJ-A8QQ	Alive	2056	24	female	M0	N0	T1b2
TCGA-C5-A1ME	Alive	1756	40	female	M0	N0	T1b1
TCGA-VS-A9UQ	Alive	1263	32	female	MX	N0	T1b
TCGA-EK-A2PL	Alive	13	36	female	NA	NA	NA
TCGA-EA-A410	Alive	803	51	female	M0	N0	T2a2
TCGA-DS-A0VN	Alive	3609	47	female	M0	N0	T1b2
TCGA-EK-A2IP	Alive	202	28	female	MX	N0	T1b1
TCGA-IR-A3L7	Alive	4483	37	female	M0	N0	T1b1
TCGA-EA-A3HU	Alive	1013	43	female	M0	N0	T2a2
TCGA-DG-A2KK	Alive	2496	51	female	M0	N1	T1b1

TCGA-WL-A834	Alive	791	57	female	NA	N1	T1b1
TCGA-ZX-AA5X	Alive	119	64	female	MX	NX	TX
TCGA-EX-A8YF	Alive	473	44	female	M0	N0	T2a1
TCGA-GH-A9DA	Alive	540	27	female	MX	N0	T1b
TCGA-Q1-A73O	Alive	428	37	female	MX	NX	TX
TCGA-EA-A5O9	Alive	788	39	female	M0	N0	T1b2
TCGA-VS-A9UP	Alive	1444	43	female	MX	NX	T2a
TCGA-EX-A69L	Alive	602	41	female	M0	N0	T1b1
TCGA-FU-A3TQ	Alive	795	55	female	MX	N1	T2b
TCGA-VS-A8EI	Alive	729	38	female	M0	NX	T3b
TCGA-VS-A9UU	Alive	442	42	female	MX	NX	T2b
TCGA-Q1-A73P	Alive	483	45	female	MX	N0	T1b
TCGA-EA-A5ZE	Alive	829	54	female	M0	N0	T1b1
TCGA-IR-A3LL	Alive	1106	60	female	M0	N0	T1b1
TCGA-JX-A3Q0	Alive	6375	63	female	M0	N0	T3
TCGA-Q1-A73Q	Alive	284	46	female	MX	NX	TX
TCGA-XS-A8TJ	Alive	890	41	female	M0	N1	T1b1
TCGA-HM-A3JK	Alive	632	64	female	MX	N1	T2b
TCGA-C5-A1BI	Alive	1112	31	female	NA	NA	NA
TCGA-EA-A556	Alive	453	38	female	M0	N0	T1b1
TCGA-EK-A2R7	Alive	27	45	female	M0	N1	T1b1
TCGA-EA-A1QS	Alive	1203	46	female	M0	N0	T1b1
TCGA-ZJ-AAXD	Alive	0	35	female	M0	N0	T3b
TCGA-Q1-A5R2	Alive	499	64	female	MX	NX	TX
TCGA-Q1-A6DW	Alive	534	44	female	MX	NX	TX
TCGA-VS-A9UY	Dead	555	29	female	MX	NX	T1b2
TCGA-FU-A3NI	Dead	638	45	female	MX	N1	T1b1
TCGA-VS-A9UV	Dead	104	74	female	MX	NX	T4
TCGA-VS-A9V5	Dead	494	50	female	MX	NX	T2b
TCGA-VS-A9UM	Dead	829	39	female	MX	NX	T3b
TCGA-UC-A7PF	Dead	2859	44	female	M0	N1	T1b1
TCGA-EA-A50E	Dead	227	45	female	M0	N1	T4
TCGA-C5-A8YT	Dead	633	36	female	NA	NA	NA
TCGA-VS-A8EB	Dead	305	41	female	M1	NX	T3b
TCGA-VS-A8EJ	Dead	607	60	female	M1	NX	T2b

TCGA-VS-A8QH	Dead	1210	76	female	MX	NX	TX
TCGA-C5-A1MJ	Dead	14	61	female	MX	N0	T1b1
TCGA-DS-A7WI	Dead	252	43	female	MX	N1	T2a2
TCGA-C5-A7CJ	Dead	3097	42	female	MX	N1	T2a
TCGA-C5-A2M2	Dead	1011	56	female	NA	NA	NA
TCGA-UC-A7PD	Dead	355	21	female	M0	N0	T1b
TCGA-HM-A3JJ	Dead	659	40	female	MX	N1	T1b1
TCGA-C5-A1BQ	Dead	604	65	female	NA	NA	NA
TCGA-C5-A1BN	Dead	166	26	female	NA	NA	NA
TCGA-EK-A2RL	Dead	1453	32	female	MX	N1	T1b1
TCGA-IR-A3LB	Dead	2032	53	female	M0	N0	T1b1
TCGA-JX-A3PZ	Dead	642	25	female	M0	N0	T1b
TCGA-JW-A852	Dead	252	42	female	MX	N1	T2b
TCGA-VS-A8EK	Dead	829	65	female	M0	NX	T4
TCGA-C5-A1MN	Dead	1245	42	female	NA	NA	NA
TCGA-VS-A94X	Dead	506	40	female	MX	NX	T2b
TCGA-VS-A8QM	Dead	951	47	female	M1	NX	TX
TCGA-DS-A1O9	Dead	266	75	female	M0	N0	T4
TCGA-C5-A8YR	Dead	837	56	female	M0	N0	T1b
TCGA-C5-A0TN	Dead	348	21	female	MX	N1	T1b
TCGA-C5-A7CK	Dead	4086	58	female	NA	NA	NA
TCGA-C5-A2LZ	Dead	3046	65	female	NA	NA	NA
TCGA-DS-A1OA	Dead	879	77	female	M0	N1	T1b1
TCGA-C5-A7CL	Dead	471	48	female	NA	NA	NA
TCGA-EX-A69M	Dead	253	62	female	M0	N0	T1b2
TCGA-JW-A5VH	Dead	100	53	female	M1	NX	T4
TCGA-C5-A1BM	Dead	2520	78	female	MX	N1	T2
TCGA-Q1-A6DT	Dead	275	55	female	MX	NX	TX
TCGA-C5-A1M9	Dead	1065	46	female	M0	N1	T3b
TCGA-HG-A2PA	Dead	773	38	female	M0	N0	T1b2
TCGA-C5-A8YQ	Dead	715	79	female	NA	NA	NA
TCGA-VS-A9V1	Dead	157	46	female	MX	NX	T3b
TCGA-C5-A1BF	Dead	570	46	female	MX	N0	TX
TCGA-C5-A1ML	Dead	636	49	female	NA	NA	NA
TCGA-VS-A9UL	Dead	442	79	female	MX	NX	T3b

TCGA-C5-A7UC	Dead	523	48	female	M0	N0	T1b
TCGA-C5-A8XH	Dead	1394	39	female	MX	N0	T1b1
TCGA-VS-A9UJ	Dead	52	55	female	MX	NX	T2b
TCGA-VS-A8QC	Dead	350	51	female	MX	NX	TX
TCGA-VS-A9V4	Dead	132	63	female	MX	NX	T4
TCGA-C5-A1MH	Dead	1186	71	female	NA	NA	NA
TCGA-C5-A3HF	Dead	543	24	female	NA	NA	NA
TCGA-VS-A9UI	Dead	1372	76	female	MX	NX	T2b
TCGA-VS-A94Y	Dead	144	47	female	MX	NX	T2b
TCGA-C5-A7X5	Dead	414	72	female	NA	NA	NA
TCGA-IR-A3LK	Dead	908	69	female	M0	N0	T1b2
TCGA-C5-A1MI	Dead	1083	50	female	M0	N1	T1b1
TCGA-DS-A0VK	Dead	1118	45	female	M0	N1	T1b1
TCGA-C5-A1M5	Dead	2052	53	female	M0	N0	T1b
TCGA-VS-A8Q8	Dead	978	26	female	MX	N0	T1b
TCGA-VS-AA62	Dead	469	51	female	MX	NX	T2b
TCGA-DS-A7WF	Dead	492	41	female	MX	N0	T1b2
TCGA-C5-A7UI	Dead	2888	42	female	MX	N0	T1b1
TCGA-C5-A1BE	Dead	2094	64	female	MX	N1	T1b
TCGA-DS-A1OB	Dead	861	45	female	M0	N1	T1b2
TCGA-C5-A1M6	Dead	955	55	female	NA	NA	NA
TCGA-C5-A7X3	Dead	284	70	female	NA	NA	NA
TCGA-UC-A7PG	Dead	370	44	female	MX	N1	T3b
TCGA-VS-A953	Dead	477	63	female	MX	NX	T4
TCGA-C5-A1MK	Dead	74	79	female	NA	NA	NA
TCGA-DS-A0VL	Dead	1692	25	female	M0	N0	T1b1
TCGA-ZJ-A8QR	Dead	582	38	female	MX	NX	TX

Table 2 Immune infiltrating cells and factors with significant difference between normal and cancer

Type	Cell	Normal (n=3)		Cancer (n=304)	
		Mean	SD	Mean	SD
CIBERSORT	Mast cell activated	1.00	0.89	0.13	0.19
	T cell follicular helper	1.00	0.59	17.48	8.75
	B cell naive	1.00	1.14	0.25	0.47
	Macrophage M1	1.00	0.26	14.73	11.04
	T cell CD4+ memory resting	1.00	0.78	0.43	0.46
	Monocyte	1.00	0.29	0.37	0.54
CIBERSORT-ABS	Mast cell activated	1.00	1.06	0.25	0.42
	T cell follicular helper	1.00	0.72	28.12	19.67
EPIC	Endothelial cell	1.00	0.26	0.15	0.11
	uncharacterized cell	1.00	0.21	1.20	0.10
	Cancer associated fibroblast	1.00	0.96	0.32	0.48
MCPCOUNTER	Cancer associated fibroblast	1.00	0.71	0.08	0.10
	Endothelial cell	1.00	0.39	0.19	0.11
QUANTISEQ	T cell CD4+ (non-regulatory)	1.00	1.06	0.11	0.28
	Myeloid dendritic cell	1.00	0.87	0.20	0.48
	Neutrophil	1.00	0.48	2.74	1.42
XCELL	stroma score	1.00	0.12	0.05	0.06
XCELL	Cancer associated fibroblast	1.00	0.07	0.05	0.07
	Endothelial cell	1.00	0.25	0.03	0.07
	Hematopoietic stem cell	1.00	0.20	0.07	0.07
	Common myeloid progenitor	1.00	0.60	0.07	0.12
	Granulocyte-monocyte progenitor	1.00	1.16	0.05	0.18
	microenvironment score	1.00	0.09	0.22	0.20
	T cell CD4+ Th1	1.00	1.26	4.76E+16	3.55E+16
	T cell gamma delta	1.00	1.31	1.63E+16	1.23E+16
	T cell CD4+ Th2	1.00	1.73	4.96E+17	3.98E+17
